# Role of Lysosomes in Silica-Induced Inflammasome Activation and Inflammation in Absence of MARCO

**DOI:** 10.1155/2014/304180

**Published:** 2014-06-26

**Authors:** Rupa Biswas, Raymond F. Hamilton, Andrij Holian

**Affiliations:** Center for Environmental Health Sciences, Department of Biomedical and Pharmaceutical Sciences, University of Montana, Missoula, MT 59812, USA

## Abstract

MARCO is the predominant scavenger receptor for recognition and binding of silica particles by alveolar macrophages (AM). Previously, it was shown that mice null for MARCO have a greater inflammatory response to silica, but the mechanism was not described. The aim of this study was to determine the relationship between MARCO and NLRP3 inflammasome activity. Silica increased NLRP3 inflammasome activation and release of the proinflammatory cytokine, IL-1*β*, to a greater extent in MARCO^−/−^ AM compared to wild type (WT) AM. Furthermore, in MARCO^−/−^ AM there was greater cathepsin B release from phagolysosomes, Caspase-1 activation, and acid sphingomyelinase activity compared to WT AM, supporting the critical role played by lysosomal membrane permeabilization (LMP) in triggering silica-induced inflammation. The difference in sensitivity to LMP appears to be in cholesterol recycling since increasing cholesterol in AM by treatment with U18666A decreased silica-induced NLRP3 inflammasome activation, and cells lacking MARCO were less able to sequester cholesterol following silica treatment. Taken together, these results demonstrate that MARCO contributes to normal cholesterol uptake in macrophages; therefore, in the absence of MARCO, macrophages are more susceptible to a greater inflammatory response by particulates known to cause NLRP3 inflammasome activation and the effect is due to increased LMP.

## 1. Introduction

Silica is a basic component of soil, sand, and most types of rocks. Silica forms a tetrahedral structure and there are two common forms of silica—crystalline silica and amorphous silica. Crystalline silica has a regular tetrahedral structure, while amorphous silica has an irregular structural arrangement. Environmental or occupational exposure to crystalline silica particles over an extended period of time results in pulmonary inflammation, which plays a vital role in pathological development of silicosis. Silicosis is a lung disease characterized by inflammation and fibrosis and remains a prevalent health problem throughout the world. Currently, treatment choices for silicosis are limited and at present no cure exists for silicosis [1, 2]. Consequently, it is important to further define mechanisms of silica-induced inflammation on which new therapeutic approaches could be developed.

Inhaled silica particles are encountered by alveolar macrophages (AM) in the lungs. The AM are the primary innate immune phagocytic cells at the air tissue interface responsible for clearance of particles through the mucociliary escalator and/or lymphatic systems [2]. Previous studies have demonstrated that AM recognize and bind silica particles through class A scavenger receptors (SR) expressed on their surface [3–6]. The class A SR family includes SR-A and MARCO, which function primarily as phagocytic receptors binding to a variety of microbial components and can also modulate inflammatory signaling by Toll-like receptors [7, 8]. SR-AI, SR-AII, and MARCO are associated with silica binding, and MARCO is the predominant receptor for binding and uptake of unopsonized particles such as silica [5, 9–13].

Previous* in vivo* results showed an increased inflammatory response in MARCO^−/−^ mice compared with WT mice following 24 hrs of silica exposure [14]. There was an increase in total protein levels and total number of lavage cells and a significant increase in infiltration of immune cells such as AM, DC, and neutrophils in MARCO^−/−^ mice compared with WT mice, all indicating an increase in inflammation in MARCO^−/−^ mice. However, the mechanism to explain the increased inflammatory response in the absence of MARCO was not clear.

Recent advances in understanding the NLRP3 inflammasome have explained the role of this pathway in inflammation and fibrosis. Particles phagocytized by AM are confined to intracellular vesicles called phagosomes, which undergo a series of interactions with endosomes and lysosomes [15–18]. Certain particles, such as silica, permeabilize the lysosome leading to release of lysosomal enzymes, including cathepsin B, to the cytoplasm [19, 20]. This triggers the assembly of the NLRP3 inflammasome—a multiprotein complex [21]. The inflammasome assembly consists of NALP3 protein, the adaptor protein ASC (apoptosis—associated speck-like protein containing a Caspase recruitment domain (CARD)), and pro-Caspase-1 [22, 23]. Adaptor protein ASC connects a NALP3 protein to pro-Caspase-1 leading to cleavage of the CARD domain of pro-Caspase-1 resulting in activation of Caspase-1. At this point another signal is required, which is triggered by endotoxins like LPS to activate the NF-*κ*B pathway. NF-*κ*B, a transcription factor, is responsible for formation of pro IL-1*β* and pro IL-18 [24]. Active Caspase-1 catalyzes cleavage of the inactive precursor molecules pro IL-1*β* and IL-18 to their active forms, IL-1*β* and IL-18 [24, 25]. IL-1*β* and IL-18 have been associated with inflammation and it is evident from the literature that inflammation and fibrosis development are closely linked to the maturation and release of these inflammasome cytokines [26–30].

We propose that the MARCO receptor can modulate the sphingomyelin pathway, leading to the lysosomal accumulation of sphingomyelin, phosphatidylcholine, and cholesterol and a decrease in ceramide. The sphingomyelin pathway is a ubiquitous signaling system, which activates multiple signal transduction pathways associated with both physiological and pathological processes that include cell growth, cell death, autophagy, angiogenesis, cancer, and inflammatory responses [31]. This pathway is initiated by the hydrolysis of membrane phospholipid sphingomyelin to ceramide. Increased ceramide production has been indicated in inflammation in response to a large variety of stressors [32]. It is anticipated that accumulation of cholesterol in the lysosomal membrane can contribute to lysosomal membrane stabilization and prevention of inflammasome activation and downstream inflammation [33–35]. Furthermore, we propose that in the absence of MARCO receptor there is decreased cholesterol accumulation in the lysosomal membrane making them susceptible to membrane permeabilization upon exposure to silica. In this study we examined the effect of MARCO on inflammasome activation upon exposure to silica. For this study, AM isolated from C57BL/6 wild-type (WT) and MARCO null mice were used to determine the role of MARCO in silica-induced inflammasome activation and inflammation.

## 2. Results and Discussion

### 2.1. Evaluation of the Effects of Silica on IL-1*β* Production in C57BL/6 WT and MARCO^−/−^ AM

**Figure 1:**
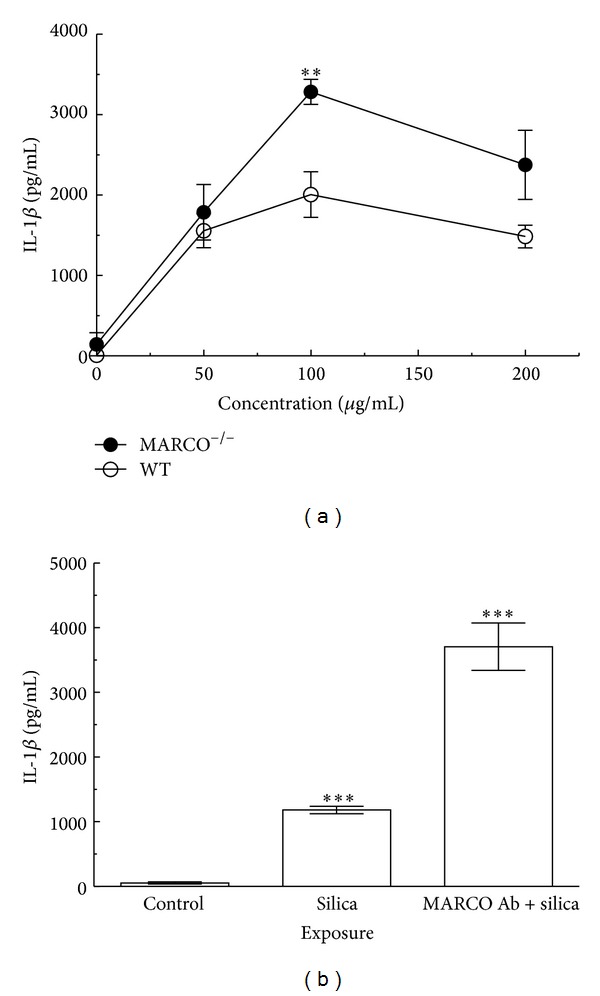
*Evaluation of the effects of doses of silica and IL-1*β* production in AM from WT and MARCO* ^−/−^ *mice.* (a) Dose response to silica-stimulated IL-1*β* release from AM cocultured with endotoxin (LPS at 20 ng/mL) 24 hrs was measured by ELISA. Mean ± SEM IL-1*β* from cultured macrophages where filled circle *⬤* indicates MARCO^−/−^ AM and open circle ⚪ indicates C57BL/6 WT AM. Double asterisk ** indicates *P* < 0.01 compared to WT control at the corresponding concentration, *n* = 4 per experimental group. (b) IL-1*β* release from WT AM with MARCO Ab. AM were pretreated with or without MARCO Ab (5 *μ*g/mL) then exposed to silica (100 *μ*g/mL) in the presence or absence of LPS (20 ng/mL) 24 hrs. Mean ± SEM IL-1*β* release from cultured macrophages was analyzed by ELISA. Triple asterisk *** indicates *P* < 0.001 compared to control at the corresponding treatment, *n* = 4 per experimental group. Unstimulated cells were used as control.

Since the molecular mechanisms involved in increased inflammation in MARCO^−/−^ compared to WT mice are not completely understood, an* in vitro* study was conducted to measure release of key cytokines and activity of key enzymes in AM of WT and MARCO^−/−^ mice. AM from MARCO^−/−^ and WT mice were stimulated with LPS (20 ng/mL) and then treated with 0, 25, 50, 100, or 200 *μ*g/mL of silica for 24 hrs. Silica caused a dose-dependent increase in IL-1*β* release from MARCO^−/−^ and WT AM. IL-1*β* release was significantly higher in MARCO^−/−^ AM than WT AM at dosage values 100 *μ*g/mL and 200 *μ*g/mL (Figure 1(a)). Maximum IL-1*β* was released at 100 *μ*g/mL and decreased at 200 *μ*g/mL in both MARCO^−/−^ and WT AM, most likely due to cytotoxicity. The increased production of IL-1*β* by MARCO^−/−^ AM relative to WT AM is consistent with the observation of increased inflammation* in vivo* in MARCO^−/−^ mice [14].

In order to determine whether the increase in IL-1*β* release from MARCO^−/−^ AM was due to the absence of MARCO receptor and not an unknown compensatory mechanism, MARCO Ab was used to block MARCO function in WT AM. IL-1*β* release from WT AM treated with LPS, silica, and MARCO Ab was also significantly higher than WT AM treated with LPS and silica only (Figure 1(b)). Furthermore, the resulting levels of IL-1*β* release by blocking MARCO receptor function using MARCO Ab with WT AM were similar to levels of IL-1*β* release from MARCO^−/−^ AM treated with LPS and silica. This result implies that the difference in IL-1*β* release between the WT AM and MARCO^−/−^ AM can be attributed to the absence of MARCO function.

### 2.2. Effect of Silica on Cathepsin B Activation

**Figure 2:**
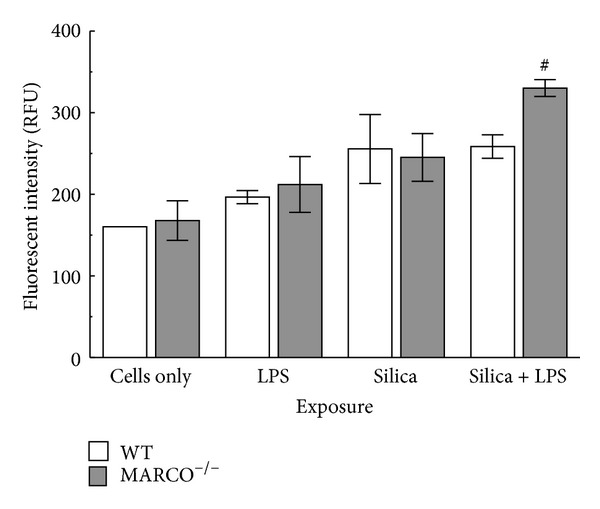
*Cathepsin B activation in MARCO* ^−/−^ *and WT AM exposed to silica.* MARCO^−/−^ AM and WT AM were stimulated with or without LPS (20 ng/mL) and then exposed to silica (100 *μ*g/mL) for 2 hrs prior to cathepsin B activity assay. Mean ± SEM cathepsin B activity where white bar indicates WT AM and grey bar indicates MARCO^−/−^ AM. Hashtag ^#^ indicates *P* < 0.05 compared to MARCO^−/−^ unstimulated (cells only) group.

Lysosomal membrane permeabilization (LMP) leads to cathepsin B release from the lysosomal lumen to the cytoplasm [19, 20]. Release of cathepsin B to the cytosol triggers NLRP3 inflammasome assembly and subsequent downstream inflammation. In order to determine whether the effect of MARCO was at LMP, the contribution of silica on cathepsin B release to the cytosol was determined (Figure 2). Primary AM from MARCO^−/−^ and WT mice were stimulated with or without LPS and then treated with silica (100 *μ*g/mL) for 2 hrs prior to cathepsin B assay. Cathepsin B activation was higher in AM treated with silica alone or silica plus LPS treated AM from MARCO^−/−^ mice compared to WT AM. The results indicate that silica exposure increases cathepsin B activity and corresponds to increased lysosomal membrane permeabilization, which was increased in the absence of MARCO.

### 2.3. Effect of MARCO on IL-1*β*

**Figure 3:**
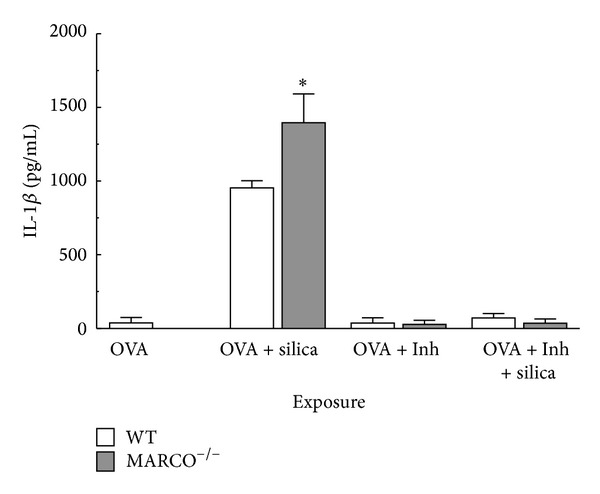
*Effect of MARCO on NLRP3 activation using IL-1*β* as an indicator* and* OVA as signal 1.* IL-1*β* release from WT and MARCO^−/−^ AM in response to silica (100 *μ*g/mL) cocultured with OVA (10 mg/mL) and with or without cathepsin B inhibitor (10 *μ*M CA-074-Me). Mean ± SEM IL-1*β* from cultured macrophages where white bars indicate WT AM and grey bars indicate MARCO^−/−^ AM. Asterisk * indicates *P* < 0.05 compared to WT, *n* = 3 per experimental group.

The activation of NLRP3 inflammasome is known to be essential for maturation of IL-1*β* [29] and cathepsin B has been proposed to work upstream of NLRP3 inflammasome assembly [36]. Therefore, the contribution of cathepsin B to silica-induced IL-1*β* release from AM was examined. As expected, AM pretreated with LPS and a cathepsin B inhibitor (10 *μ*M CA-074-Me added 30 min prior to silica exposure) showed a significant reduction in IL-1*β* release compared to the absence of the cathepsin B inhibitor (Figure 3). The MARCO receptor is known to bind LPS [3], and in the absence of MARCO, more LPS could be expected to bind to CD14 and signal more pro-IL-1*β* production [37, 38]. In order to rule out the possibility that the differences in IL-1*β* release in the absence of MARCO were due to increased availability of LPS for signaling through CD14, ovalbumin (OVA) was used as an alternate stimulant to increase pro-IL-1*β* levels in AM [39]. As shown in Figure 3, IL-1*β* release with OVA as a stimulant was comparable to LPS. Furthermore, CA-074-Me was effective in blocking IL-1*β* release. Therefore, the results with OVA and the cathepsin B inhibitor indicate that the difference in IL-1*β* maturation in MARCO^−/−^ and WT was not due to differences in availability of LPS acting on CD14 and that regardless of the source of signal 1 (LPS or OVA) inhibiting cathepsin B blocked IL-1*β* maturation and release.

### 2.4. Effect of Silica on Caspase-1

**Figure 4:**
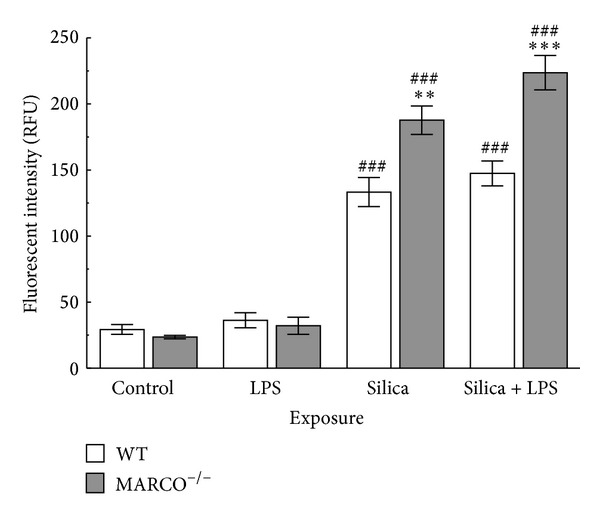
*Caspase-1 activation in MARCO* ^−/−^ *and WT AM exposed to silica.* Caspase-1 activation in WT and MARCO^−/−^ AM was measured 4 hrs after treatment with silica (100 *μ*g/mL) with or without LPS (20 ng/mL) as described in Methods. Mean ± SEM white bars indicate WT AM and grey bars indicate MARCO^−/−^ AM. Double asterisk ** indicates *P* < 0.01 and triple asterisk *** indicates *P* < 0.001 compared to WT at corresponding treatment. ^##^ indicates *P* < 0.01 compared to control in the same mice strain. *n* = 3 per experimental group.

Measuring IL-1*β* release is a proxy measure of NLRP3 inflammasome activation since it requires both signal 1 (NF-*κ*B mediated pro-IL-1*β* generation) and signal 2 (NLRP3 inflammasome activation). Therefore, a more direct assay for NLRP3 inflammasome activation is to measure Caspase-1 activity. Primary AM from MARCO^−/−^ and WT were stimulated with or without LPS and then treated with silica (100 *μ*g/mL) for 4 hrs prior to Caspase-1 assay. The pattern of results for Caspase-1 activity was similar to IL-1*β* release in that it was higher in MARCO^−/−^ AM compared to WT AM (Figure 4). Therefore, the results suggest that the effect of MARCO on the NLRP3 inflammasome or signal 2 pathway does not involve signal 1, consistent with the above findings using OVA as a signal 1 initiator. Furthermore, the findings are consistent with the cathepsin B results, which also suggested the effect of MARCO on modulating the NLRP3 inflammasome pathway. Taken together, the results suggest that the target of the contribution of MARCO in regulating IL-1*β* release with silica may well be at lysosomal membrane permeability since in the absence of MARCO there is less silica being taken up although there is more cathepsin B release and correspondingly greater Caspase-1 activity.

### 2.5. Induction of Pro-IL-1*β* Expression in AM

**Figure 5:**
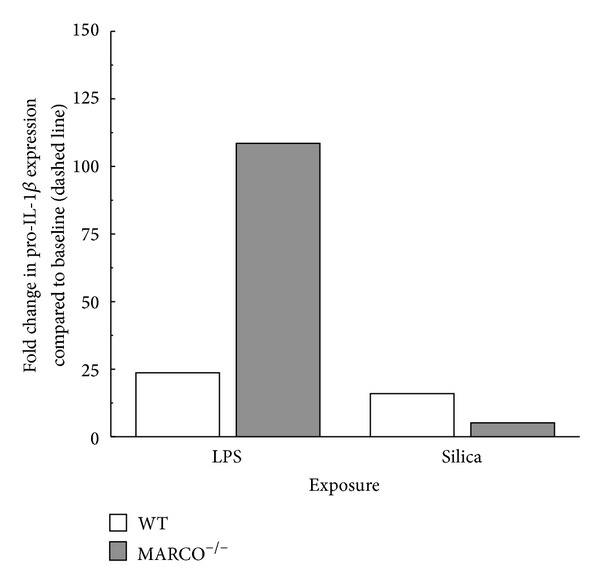
*Pro-IL-1*β* mRNA expression in WT and MARCO* ^−/−^ *AM analyzed using RT-PCR.* White bars indicate WT AM and grey bars indicate MARCO^−/−^ AM. Fold change in pro-IL-1*β* expression was compared to unstimulated control. The data represent one experiment with 2 replications.

Pro-IL-1*β* mRNA expression in MARCO^−/−^ and WT AM was measured in order to evaluate the contribution of MARCO to signal 1 activation. Primary AM from MARCO^−/−^ and WT mice were treated with LPS, silica, or left untreated. Pro-IL-1*β* expression in AM was measured by RTPCR and the results are shown in Figure 5. Pro-IL-1*β* expression was higher in MARCO^−/−^ AM compared to WT following LPS stimulation. This finding is consistent with the increased binding of LPS to CD14 in the absence of MARCO, although as the results presented in Figure 4 illustrated that primary contribution of MARCO was to regulate signal 2. Silica treatment produced negligible expression of pro-IL-1*β* in MARCO^−/−^ and WT AM tested. These results confirm that LPS is a stimulant for pro-IL-1*β* expression, while silica exposure has minimal to no effect on pro-IL-1*β* expression. Consequently, activation of signal 2 has little impact on signal 1.

### 2.6. Effect of Inflammasome Activation on IL-6 Release

**Figure 6:**
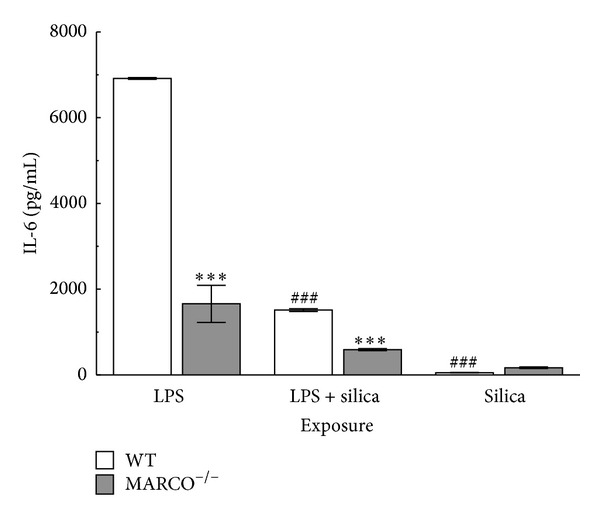
*Effect of inflammasome activation on IL-6 release.* IL-6 release from WT and MARCO^−/−^ AM in response to silica (100 *μ*g/mL), LPS (20 ng/mL), or silica and LPS. Mean ± SEM IL-6 from cultured AM where white bars indicate WT AM and grey bars indicate MARCO^−/−^ AM. Triple asterisk *** indicates *P* < 0.001 compared to WT at corresponding treatment, ^###^ indicates *P* < 0.001 compared to control in the corresponding mouse strain, *n* = 3 per experimental group.

In order to evaluate whether the effect of MARCO was specific (restricted to inflammasome cytokines) or more global on macrophage cytokine production, the effect of silica on MARCO^−/−^ and WT AM IL-6 production, which does not require NLRP3 inflammasome activation, was also measured after 24 hrs (Figure 6). In contrast to the IL-1*β* production results, IL-6 levels in the LPS-treated group in MARCO^−/−^ AM were significantly lower than WT AM. There was also significantly less IL-6 in the LPS- and silica-treated groups, compared to LPS stimulated group. These results are consistent with the known ability of LPS to stimulate IL-6 production and IL-6 production does not depend on inflammasome activation. However, IL-6 production is not upregulated by MARCO.

### 2.7. Effect of Silica on Acid Sphingomyelinase Activity

**Figure 7:**
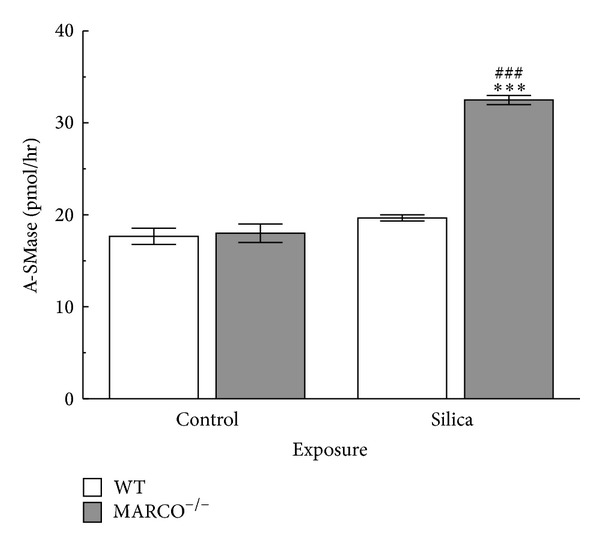
*Effect of silica on acid sphingomyelinase activity.* Acid sphingomyelinase activity in WT and MARCO^−/−^ AM, 1 hr after treatment with silica (100 *μ*g/mL). White bars indicate WT AM and grey bars indicate MARCO^−/−^ AM. Triple asterisk *** indicates *P* < 0.001 compared to WT at corresponding treatment. ^###^ indicates *P* < 0.001 compared to control at the corresponding mouse strain, *n* = 3 per experimental group.

Acid sphingomyelinase is a lipid-metabolizing enzyme localized in lysosomes [32]. Acid sphingomyelinase hydrolyses membrane phospholipid sphingomyelin to ceramide [31]. In addition acid sphingomyelinase is an important regulator of the sphingolipid metabolism pathway [40]. Acid sphingomyelinase levels were measured following silica treatment of AM because of its reported association with inflammation and acute lung injury [41]. Primary AM from MARCO^−/−^ and WT mice were treated with or without silica (100 *μ*g/mL) for 1 hr. After 1 hr cell lysates were prepared for sphingomyelinase activity and the results are shown in Figure 7. There was a significant increase in acid sphingomyelinase activity in MARCO^−/−^ AM treated with silica compared to WT AM incubated with silica. These results indicate that acid sphingomyelinase activity was significantly higher in MARCO^−/−^ AM than WT supporting the notion that silica exposure affects the sphingolipid pathway and consequently may decrease sphingomyelin and increase ceramide levels in the absence of MARCO [32].

### 2.8. Effect of Silica on Intracellular Cholesterol

**Figure 8:**
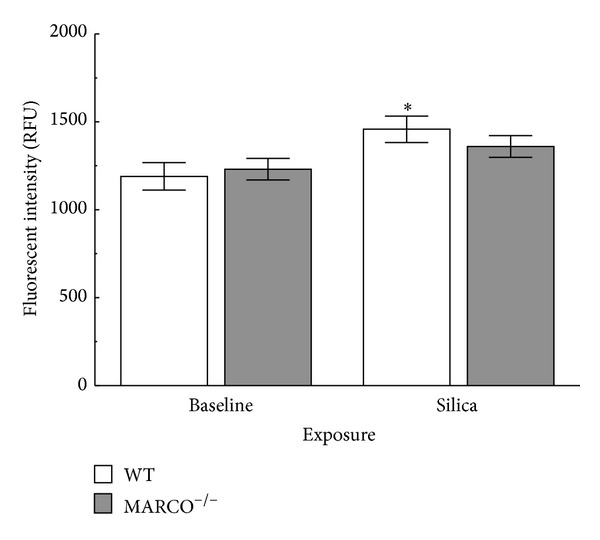
*Increased cholesterol uptake in WT AM in response to silica*. Isolated AM were loaded with TopFluor cholesterol (1 *μ*g/mL) for 24 hrs prior to silica (50 *μ*g/mL) exposure in RPMI media with 5% delipidated FBS. Silica exposure was 4 hrs in regular RPMI culture media. The wells were read in a microplate reader at 488 nm ex/525 nm em. Asterisk * indicates statistical significance at *P* < 0.05 compared to baseline WT response, *n* = 7.

The results above demonstrated that silica exposure impacted lipid metabolism, which is associated with lysosomes. Since the critical impact of silica signaling appears to be on lysosomal membrane permeability and cholesterol is known to affect lysosomal integrity [33] the potential role of cholesterol content in AM was examined as a potential mechanistic explanation for LMP. Therefore, cholesterol levels in WT and MARCO^−/−^ AM were assessed by loading cells with 1 *μ*g/mL TopFuor cholesterol for 24 h and then treated with or without silica for 4 hrs. As expected, there is very large background fluorescence due to the naturally high cholesterol content in AM (Figure 8). There was a significant increase in intracellular cholesterol (represented as an increase in TopFuor fluorescence) in WT AM in response to silica exposure compared to control. This outcome is consistent with the proposal that silica is inducing cholesterol uptake and could contribute to protection against LMP. However, intracellular cholesterol in MARCO^−/−^ AM in response to silica exposure did not change significantly compared to the baseline control and was less than that taken up by WT AM (although it was not significantly different from WT AM treated with silica). This result is consistent with the proposal that AM lacking MARCO were not able to take up as much cholesterol making them more susceptible to LMP. This would also explain why the difference in IL-1*β* release was only evident at the higher doses of silica.

### 2.9. Effect of Lysosomal Cholesterol on NLRP3 Inflammasome

**Figure 9:**
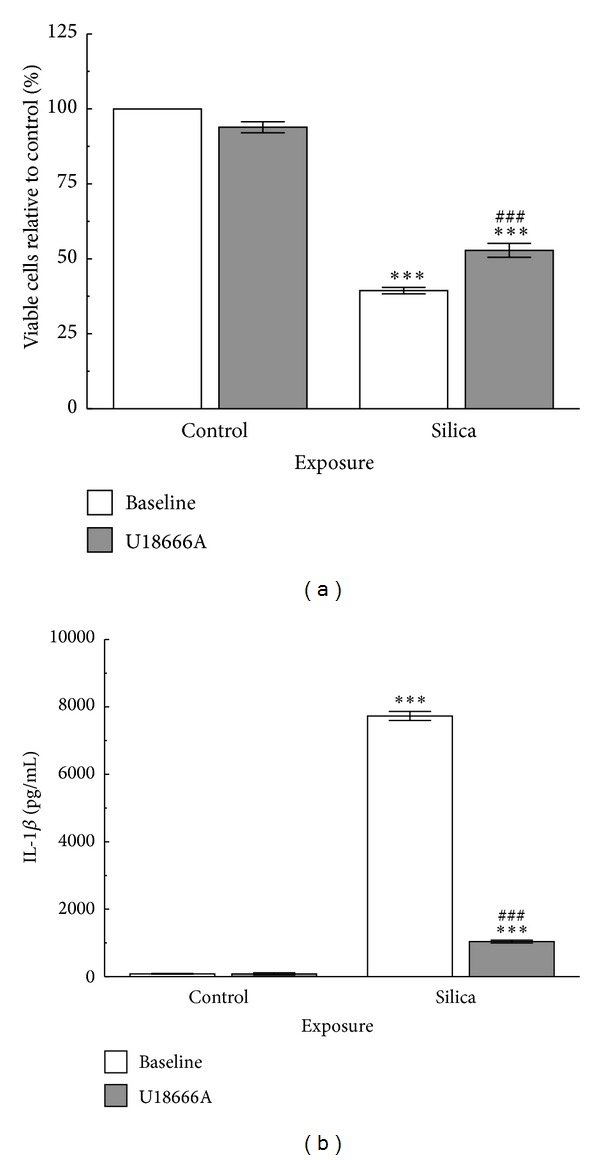
*Cholesterol trafficking modifier U18666A decreases WT AM response to silica (50 *μ*g/mL).* (a) Cell viability at 24 hrs after particle exposure by MTS assay. (b) IL-1*β* release 24 hrs following silica exposure cocultured with LPS (20 ng/mL) to stimulate NF-*κ*B activation. Asterisks *** indicate statistical significance at *P* < 0.001 compared to corresponding control condition. Hashtags ^###^ indicate significance at *P* < 0.001 compared to baseline silica-exposed response, *n* = 3.

The above results suggested that cholesterol in the lysosomal membrane can influence lysosomal membrane stabilization, inflammasome activation, and downstream inflammation. In order to confirm the role of cholesterol on silica-induced LMP leasing to IL-1*β* release, the cholesterol trafficking modifier U18666A was used. U18666A blocks the intracellular trafficking of cholesterol and the exit of free cholesterol from the late endosomal compartment [42]. It also inhibits oxidosqualene cyclase and desmosterol reductase eliminating cholesterol biosynthesis [42]. Primary AM from WT mice were treated with U18666A for 24 hrs and then exposed to silica followed by LPS for another 24 hrs. Cell viability was also measured at 24 hrs after particle exposure by the MTS assay. Silica caused a significant decrease in cell viability; however, there was significant increase in cell viability in silica-U18666A exposed group compared to baseline silica group demonstrating protection against cell death (Figure 9(a)). There was the expected significant IL-1*β* release following silica exposure (Figure 9(b)) compared to control. However, IL-1*β* release was significantly less in the silica-U18666A exposed group compared to silica treatment alone. Taken together, these results indicate that lysosomal cholesterol accumulation can decrease LMP, NLRP3 inflammasome activation, and IL-1*β* release.

## 3. Experimental Section

### 3.1. Mice

Breeding pairs of C57Bl/6 were originally purchased from the Jackson Laboratory (Bar Harbor, ME), while MARCO null breeders were kindly provided by Dr. Lester Kobzik (Harvard School of Public Health, Boston, MA). MARCO null mice were on C57BL/6 background. Animals were housed in microisolators on a 12-hr light/dark cycle. The mice were maintained on an ovalbumin-free diet and given deionized water ad libitum. Age matched (6–8 week) male and female mice were used in all studies. All mice were maintained in the University of Montana specific pathogen free laboratory animal facility. The University of Montana Institutional Animal Care and Use Committee approved all animal procedures.

### 3.2. Particles

Crystalline silica (Min-U-Sil-5, average particle size 1.5–2 *μ*m in diameter) was obtained from Pennsylvania sand glass corporation (Pittsburgh, PA) and was acid-washed in 1 M HCl at 100°C for 1 hr. The silica was washed in sterile water three times and dried in an oven at 200°C to remove all water. A stock suspension of 5 mg/mL in phosphate-buffered saline (PBS) was made. Silica suspensions were sonicated for 1 min at half max power in a Masonix cup-horn sonicator (XL2020, Farmingdale, NY) attached to a ThermoForma circulating water-bath at 550 watts and 20 Hz (8000 Joules).

### 3.3. Alveolar Macrophage Isolation and Culture

Mice were euthanized by a lethal injection of Euthasol (Virbac Corp, Fort Worth, TX). The lungs were removed with the heart. The lungs were lavaged five times with 1 mL of cold PBS. Pooled cells were centrifuged at 1500 ×g for 5 min. The lavage fluid was aspirated and discarded. The cells were resuspended in 1 mL of RPMI 1640 culture media supplement with 10% fetal bovine serum, 100 IU penicillin, and 100 *μ*g/mL streptomycin (Media tech, Inc., Herdon, VA). Total nucleated lavage cell fractions were counted by lysing the red blood cells with Zap-globin reagent II lytic reagent (Beckman Coulter, Fullerton, CA) and by using Z2 Coulter particle count and size analyzer (Beckman Coulter, Fullerton, CA). The concentration of cells was then adjusted to 10^6^ cells/mL before doing any further steps. Later the cells were treated with or without silica or endotoxin or a combination of the two. The cells were cultured in a cell culture plate overnight at 37°C in a water-jacketed CO_2_ (5%) incubator (ThermoForma, Mariette, OH). For determining specificity of MARCO in IL-1*β* release experiment, AM from WT mice were treated with MARCO Ab (polyclonal goat IgG, rmMARCO; aa 70–518 assession number: Q60754, Catalog number: AF2956 obtained from R&D Systems, Minneapolis, MN) 30 min prior to exposure to silica or endotoxin for 24 hr. IL-1*β* was measured as described below.

### 3.4. Cytokine Assays

Culture supernatants were assayed for cytokines with commercially available kits according to the manufacturer's protocol. IL-1*β* measurements were determined by using Duo-set kits (BD and R&D systems). IL-6 measurements were determined using Duo-set kits (R&D systems). Colorimetric analysis was done using a Spectra Max 190 plate reader (Molecular Devices, Sunnyvale, CA) at 450 nm. Data are expressed as mean ± SEM picograms/mL of retrieved culture supernatant.

### 3.5. RNA Isolation

RNA Isolation was performed using Trizol according to the manufacturer's protocol (Trizol Reagent, Invitrogen, Carlsbad, CA). Further purification was performed using the E.Z.N.A. Total RNA Kit I according to the RNA cleanup protocol from Omega Bio-Tek, Inc. (Norcross, GA). The final elution step was performed with 40 *μ*L of RNase free water. RNA quantity was determined using a NanoDrop ND 1000 Spectrophotometer (NanoDrop Technologies, LLC, Wilmington, DE).

cDNA production was performed according to the qScript cDNA protocol from Quanta BioSciences (qScript cDNA SuperMix, Gaithersburg, MD).

### 3.6. Real Time PCR

Pro IL-1*β* message expression was measured using a Stratagene Mx3005p instrument with MxPro software (Agilent Technologies, Santa Clara, CA). Primers (Integrated DNA Technologies, Coraville, IA, USA) were as follows: *β*-actin forward sequence (5′-ACACTGTGCCCATCTACGAG-3′), reverse sequence (5′-TCAACGTCACACTTCATGATG-3′), and IL-1*β* primers were derived from IL-1*β* sequence (accession number: NM_008361.3), forward primer sequence (GGTACATCAGCACCTCACAA), reverse primer sequence (TTAGAAACAGTCCAGCCCATAC). Reactions were performed using FastStart Universal SYBR Green Master (ROX) mix (Roche Diagnostics Corp., Indianapolis, IN) according to manufacturer's protocol. Expression levels were normalized to Actb expression, and fold changes were calculated using the ΔΔCt method.

### 3.7. Acid Sphingomyelinase Assay

Cell lysates were prepared from alveolar macrophages using a Dounce homogenizer. Cell lysates were assayed for acid sphingomyelinase with commercially available kits (K-3200 Echelon biosciences Inc., Salt Lake City, UT 84108) according to the manufacturer's protocol. Colorimetric analysis was done using a Gemini XS plate reader (Molecular Devices, Sunnyvale CA) at 360 nm excitation and 460 nm emission. Data are expressed as pmol/hour of active acid sphingomyelinase.

### 3.8. Cathepsin B Activation Assay

Isolated AM were used to determine cathepsin B activation. Cathepsin B activation was determined in the AM after 2 hr of incubation with silica, silica and LPS, LPS and unstimulated cells. Cathepsin B activity was determined using Magic Red cathepsin B Detection Kit (ImmunoChemistry Technologies, Bloomington, MN 55431). The Gemini XS plate reader was used to analyze cathepsin B activation at 590 ex and 630 em (Molecular Devices, Sunnyvale CA).

### 3.9. Caspase-1 Activation Assay

Caspase-1 activation was determined in isolated AM after 4 hr of incubation with silica, silica and LPS, LPS and nontreated cells. Caspase-1 activity was determined using FAM-FLICA* in vitro* Caspase-1 kit (ImmunoChemistry Technologies, LLC, Bloomington, MN 55431). The Gemini XS plate reader was used to analyze Caspase-1 activation at 492 ex and 520 em (Molecular Devices, Sunnyvale, CA).

### 3.10. TopFluor Cholesterol Loading Experiments

Isolated AM from C57BL/6 WT and MARCO^−/−^ mice (process described above) were cultured at 100 × 10^3^ cells per well in 96-well tissue culture plates for 24 hrs prior to silica exposure. The cells were cultured with 1 *μ*g/mL TopFuor cholesterol (Avanti Polar Lipids, Alabaster, AL) in RPMI media with 5% delipidated FBS. After the 24 hr loading period, the cells were switched to normal media described above and exposed to silica at 50 *μ*g/mL for 4 hr. The plates were read in a Gemini fluorometric plate reader (Molecular Devices, Sunnyvale CA) at 488 ex and 525 em.

### 3.11. U18666A Cholesterol Modification Experiments

Isolated AM from C57BL/6 WT mice (process described above) were cultured at 100 × 10^3^ cells per well in 96-well tissue culture plates for 24 hr ± 1 *μ*M U18666A (Sigma, St Louis, MO) prior to silica exposure. U18666A is an amphipathic steroid 3-*β*-[2-(diethylamine)ethoxy] androstenone. It blocks the intracellular trafficking of cholesterol and the exit of free cholesterol from the late endosomal compartment. The mechanism of action for the accumulation of cholesterol in late endosomes and lysosomes can be attributed to the amphipathic property of the compound [42]. U18666A also inhibits oxidosqualene cyclase and desmosterol reductase eliminating cholesterol biosynthesis [42]. After the 24 hr loading period, the cells were exposed to silica at 50 *μ*g/mL, in addition to a 20 ng/mL LPS coculture, for another 24 hr. The LPS was used to stimulate NF-*κ*B, leading to pro-IL-1*β* production. The culture media was retrieved for IL-1*β* assay (R&D Systems, Minneapolis MN) and the cells were tested for viability using a MTS assay according to the manufacturer's instructions (Promega, Madison, WI).

### 3.12. Statistical Analysis

Statistical analyses involved comparison of means using a one- or two-way* ANOVA* followed by Dunnett's test or Bonferroni's test to compensate for increased type I error. All probabilities were two-tailed unless otherwise stated. Statistical power was greater than 0.8. Statistical significance was defined as a probability of type I error occurring at less than 5% (*P* < 0.05). The minimum number of experimental replications was 3-4 depending on the experiment. Graphics and analyses were performed on PRISM 6.0.

## 4. Conclusions

This study focused on understanding the role of MARCO in inflammation on silica exposure. In the present study we demonstrated that in the absence of MARCO receptor or when MARCO Ab was used to block MARCO receptor function, silica increased NLRP3 inflammasome activation and proinflammatory cytokine release in AM from MARCO^−/−^ mice compared to AM from WT mice. Exposure to silica caused more LMP and greater cathepsin B release in MARCO^−/−^ AM compared to WT AM. These results suggested that the absence of MARCO enhanced AM susceptibility to silica at the level of LMP. The mechanism of the increased LMP in the absence of MARCO was attributed to changes in cholesterol trafficking. In WT AM silica treatment resulted in increased cholesterol uptake that was greatly reduced in the absence of MARCO. Furthermore, blocking cholesterol trafficking with U186666A greatly decreased silica-induced cytotoxicity and inflammasome activation. Taken together, these results demonstrate the important role that cholesterol plays in phagolysosomal stability and suggest a potential therapeutic site to decrease particle-induced inflammation.
